# Human Papillomavirus types prevalence and their association with cervical dysplasia among HIV and non-HIV infected women attending reproductive health clinics in Eastern Kenya

**DOI:** 10.4314/ahs.v22i1.14

**Published:** 2022-03

**Authors:** Njue James Kinotia, Margaret Muturib, Lucy Kamauc, Raphael Lwembed

**Affiliations:** 1 Department of Medical Laboratory Sciences, Kenyatta University; 2 Centre for Virus Research, Kenya Medical Research Institute (KEMRI)

**Keywords:** Human Papillomavirus, cervical dysplasia, HIV, Eastern Kenya

## Abstract

**Background:**

Human Papillomavirus (HPV) causes over 99% of all cervical cancer globally. In 2019; it was responsible for 3,286 deaths in Kenya. Understanding the epidemiological distribution of HPV genotypes by cervical dysplasia and HIV infection is important in designing prevention strategy and management of cervical cancer.

**Objective:**

To determine HPV genotypes prevalence and their distribution by cervical dysplasia, social-demographic and risk factors associated with cervical cancer among HIV-infected women aged 18–48 years seeking reproductive healthcare in Eastern Kenya.

**Methods:**

Cervical specimens were obtained for cytology, HPV-genotyping, histology while social-demographic factors were collected using a questionnaire and analysed using Pearson chi-square test.

**Results:**

317 womencases: 161(50.8%); control 156(49.2%), mean age: 34.3, range 18–46 years were recruited. Thirteen HPV genotypes associated with cervical dysplasia were: CIN1{cases: HPV81[12(3.8%), HPV11[2(0.6%); control: HPV53 and HPV66[1(0.3%)}, CIN2 {cases: HPV11, HPV16, HPV661(0.3%), HPV816(1.9%) and single case1(0.3%) of HPV9, HPV11, HPV16, HPV44, HPV66, HPV81 HPV88, HPV53 and HPV58; control: HPV81[2(0.6%)} and invasive cancer {cases: HPV16[1(0.3%) and HPV81[3(0.9%); control: HPV16 and HPV66[1(0.3%).

**Conclusions:**

Cervical dysplasia was associated with more mixed-lr/hrHPV genotypes among HIV-infected than HIV-uninfected women. The finding adds to the pool of knowledge the epidemiological data required in determining the population at risk for cervical cancer.

## Introduction

Human Papillomavirus (HPV) is primarily responsible for 99.7% of cervical cancers globally. It is sexually-transmitted and causes cervical neoplastic changes leading to cervical cancer; the second most common type of cancer in women aged 15–44 years in Kenya. It was responsible for 311,365(8.2%) annual global mortality and 3,250(12.8%) in Kenya in 2019^1^. Cervical-screening rate in Kenya is only 3.2% for women aged over 18 years^1^,^2^. Human Papillomaviruses are grouped based on their oncogenecity as Group I: Carcinogenic: HPV16, 18, 31, 33, 35, 39, 45, 51, 52, 56, 58, and 59, Group 2A: proba bly-carcinogenic: HPV26, 53, 66, 67, 70, 73, 82, 30, 34, 69, 85 and 97, Group 2B: possibly-carcinogenic: HPV6 and 11 and Group 3: unclassifiable to carcinogenicity^3^,^4^,^5^. Their oncogenecity is increased by Human Immunodeficiency virus (HIV) infection, long-term exposure to hormonal-contraceptives which favor tumorigenic effects of HPV-genome and weakens the immune system responsible of clearing cancer cells. Other risk factors include early sex-debut and long-term inflammation caused by recurrent genital infections,^4^,^5^,^7^. This study therefore aimed at determining HPV type's prevalence and their association with cervical dysplasia by HIV-serostatus. The genotype's prevalence was determined by social demographic and risk-factors associated with HPV oncogenecity.

## Methods

### Study design and participants

This cross-sectional study involved 317 women aged 18–46 years in County Referral Hospital's (Isiolo, Kirinyaga, Meru, Tharaka-Nithi, and Embu) Reproductive Health clinics of eastern Kenya in 2019. A sample size was calculated by infection rate of 2.8%^1^ and distributed by 2017 clinic's attendance. Stratified sampling by county of residence and simple-random sampling per county were used to recruit participants.

### Social-demographic data collection

Social-demographic data on residence, age, education level, parity, sexual-orientation, and family-planning method were collected by a nurse using a translated (Swahili) questionnaire.

### HIV determination

HIV serostatus was determined using the national algorithm^8^; Base-line test (Alere Determine®HIV-1/2-Abbort), confirmatory test (First-Response® HIV1-2-Premier, Medical Corporation) and tie-breaker test (Uni-Gold™ Recombigen® HIV-1/2 by Trinity Biotech).

### Collection and storage of cervical exfoliated cell samples

External genitalia and cervical opening (os) were examined with a speculum while the participant lay in a lithotomic position. Cervical cytological specimens were collected using a cervical brush (Dacron™ cervical brush; Digene Corporation, Maryland), spread and fixed immediately on a clean glass slide. The brush was stored and transported at 1-4°C in Digene Specimen Transport Medium for HPV-genotyping.

### Cytology

Pap smears were reported by a cytopathologist using Bethesda 2014 guidelines^9^,^10^ as normal or abnormalAtypical Cells of Unknown Significance (ASCUS), Low-grade squamous intraepithelial lesion (LSIL), High-grade squamous intraepithelial lesion (HSIL), atypical squamous cells, cannot exclude HSIL (ASC-H) or Atypical glandular cells (AGC). Unknown inflammation and cervical infections (candidiasis, cervicitis, trachomatis, and bacterial vaginitis) were also reported.

### HPV DNA genotyping

The following procedures were used for HPV genotyping:

**a. DNA extraction**

A 96-well format HighPrep™ Viral-DNA/RNA, Mag-Bio Genomics, USA/Canada Lysis kit was used. Samples stored at 1-4°C were thawed, vortexed (5min utes) then centrifuged (10000r/min-5minutes) to ex tract cytological material from the brush into media.

**b. HPV DNA PCR**

HPV detection was achieved by amplifying an L1 portion of the HPV genome that is relatively conserved through nested PCR in the ABI-thermocycler Model 9600; Applied Biosystems® using HPV consensus primary primers PGMY09: GCACAGGGACATAACAATGG and PGMY11: CGTCCCAAAGGAAACTGATC targeting 450bp and secondary primers MGP5+: ACGTTGGATGTTTGTTACTGTGGTGGATACTAC) and MGP6+: ACGTTGGATGGAAAAATAAACTGTAAATCATATTCCT targeting ∼160bp in L1 genome ORF^11^,^12^,^13^,^14^. 5µM working stock of each primer was prepared by adding 50µL biotinylated PGMY09 100µM to 350µL nanopure-water and 50µL PGMY11 100µM primers to 750µL nanopure water. They were later distributed each 5µM working stock in 45–90µL aliquots and stored at -20°C.

A mastermix containing PCR buffer (1X), 2.0mM MgCl2, 100µM dNTPs, 0.13 parts Taq polymerase-enzyme, 500nM of respective forward and reverse primary and secondary primers. 5µl of the DNA extract was used in primary PCR while 5µl of primary PCR product was used in nested PCR. First reaction: 4minutes at 95°C (initial denaturation) then 30cycles of 20 seconds at 95°C, 40 seconds at 56°C then 2 minutes at 72°C. Nested reaction: 4minutes at 95°C (initial denaturation) then 30cycles of 20seconds at 95°C and 40 seconds at 60°C then extension (7 minutes) at 72°C. Positive control of known CIN2+ and negative control (distilled water) were incorporated in both reactions.

**c. Gel electrophoresis and UV visualization**

Tris-Borate-EDTA 10X was prepared by dissolving 162g Tris-base, 50g boric acid, 9.5g EDTA in 1liter nanopure water (pH8.8). 5µl PCR-product in 4% agarose was used in gel-electrophoresis^12^,^14^. The positive PCR-product was purified using the QIAquick DNA purification kit™(Qiagen, Germany).

**d. HPV DNA sequencing**

DNA sequencing was performed in ABI-thermocycler Model 9600 (Applied Biosystems) for 20 reaction cycles of 1µL positive PCR-product, 1µL of 5µM GP6+ primer, 1µLBigDye® Terminator, 3.5µL buffer (5x), 13.5ml nanopure water according to the protocol. Sep column (Princeton Separations, Adelphia, NJ) was used for dye-terminator cleanup followed by sequencing in ABI3130 four-capillary Genetic Analyzer.

**e. HPV genotyping and phylogenetic analysis**

Sequences were edited with CHROMAS software Version 2.4.3 then blasted in NCBI http://blast.ncbi.nlm.gov/blast.cgi. HPV type-sequences with unique divergence were phylogenically analysed and referenced from GenBank. Representative sequences and their references in input file underwent multiple alignments with CLUSTAL W in MEGA X software^14^. The Maximum Likelihood method and the Tamura-Nei model were used to infer evolutionary history. Neighbor-Join and BioNJ algorithms were used to construct initial trees for the heuristic search of the matrix of pair wise distances by the Maximum Composite Likelihood (MCL) method by selecting the topology with superior log likelihood value. Eighty-six nucleotide sequences were involved while codon positions were 1^st^+2^nd^+3^rd^.

### Histology

Colposcopy examination and histological analysis of biopsy tissues collected within 1–2 weeks following abnormal Pap smear results (LSIL, HSIL, ASC-H and AGC) were reported as Carcinoma-in-Situ (CIS), Invasive cervical cancer (ICC) or Cervical Intraepithelial Neoplasia (CIN); CIN1, CIN2 or CIN3 depending on the abnormal-epithelium thickness of ⅓, ⅔, or entire thickness respectively)^14^,^15^.

### Ethics approval

KEMRI Scientific Ethical Review Unit approved the study (KEMRI/SERU/CVR/004/3342). Participants were consented orally and data collected was confidentially stored by the Principle Investigator. Mentally-incompetent participants were excluded.

## Results

**a. Distribution of HPV genotypes among HIV and non-HIV infected women (n=106).**

**Figure 1:**
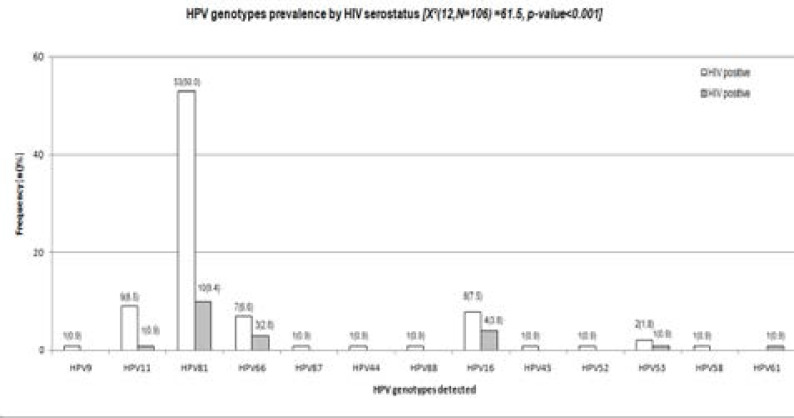
Association between HPY types detected and HIV serostatus

Thirteen HPV types detected were low-risk HPV9, HPV11, HPV81, HPV66, HPV87, HPV88, high-risk HPV16, HPV53, HPV61, HPV45, HPV52, and HPV58 (p<0.001) (Figure 1).

**b. Distribution of low/and high-risk HPV genotypes as single on multiple infection among HIV and non-HIV infected women**

A total of 62(19.2%) HIV-infected women had single HPV type infection compared to (93.2%) HIV-uninfected women while 73(23.03%) were infected by multiple HPV types compared with 13(4.1%) HIV-uninfected women (p<0.001) (Table 1).

**Table 1:** Total HPV genotypes prevalence identified as single or multiple infections

Infection type	N(%)	HIV negative	HIV positive	p-value
**Single HPV type infection**				
low-risk type	67(21.1)	9(2.8): HPV 81	7(2.2): HPV11 51(16.1): HPV81	<0.001**
high-risk type	4(1.3)		3(0.1): HPV16 1(0.3): HPV66	
Total (single HPV types)	**71(22.4)**	**9(3.2)**	**62(19.2)**	
**Multiple HPV types of infection**				
low-risk type	2(0.6)		1(0.3): HPV81,44 1(0.3): HPV81,88	
high-risk type	9(2.8)	3(0.9): HPV16, 66	4(1.3): HPV16, 66 1(0.3): HPV53,66 1(0.3): HPV16,58	
low and high-risk type	4(1.2)	1(0.3): HPV11,16,53, 61, 81	1(0.3): HPV9,53 1(0.3): HPV11,66 1(0.3): HPV11,45,52,87	
**Total (multiple HPV types)**	**15(4.7)**	**4(1.2)**	**11(3.5)**	
**Total (N=317)**	**86(27.12)**	**13(4.1)**	**73(23.03)**	

** the probability at the 0.001 level

**c. HPV prevalence by social demographic and HPV-associated risk factors among HIV and non-HIV infected women**

A total of 317mean age: 34.3years, SD±10.4, range: 18–46 women were recruited. Overall HPV prevalence was 27.1%[(86/317); cases: 23.2%(73/317); control: 4.1%(13/317). There was significant association between HPV infection and age, parity, education level and reported number of sex partners (Table 2).

**Table 2:** HPV infection-rate by socio-demographic and HPV associated risk factors among HIV and non HIV-infected women

Category	N(%)	Total HPV prevalence	HIV negative [n(%)]				HIV positive [n(%)]			

			HPV status		Total	p- value	HPV status		Total	p- value
	
			Negative	positive			negative	positive		
Residence										
Embu	85(26.8)	27(8.5)	36(11.4)	5(1.6)	41(13.0)	0.853	22(6.9)	22(6.9)	44(13.8)	0.383
Isiolo	64(20.2)	18(5.7)	35(11.0)	3(0.9)	38(11.9)		11(3.5)	15(4.7)	26(8.2)	
Kirinyaga	56(17.7)	12(3.8)	22(6.9)	1(0.3)	23(7.2)		22(6.9)	11(3.5)	33(10.4)	
Meru	81(25.6)	20(6.3)	37(11.7)	3(0.9)	40(12.6)		24(7.6)	17(5.4)	41(13.0)	
T.Nithi	31(9.8)	9(2.8)	13(4.1)	1(0.3)	14(4.4)		9(2.8)	5)	17(5.3)	

**Age [mean: 34.3, range 18–48]**										
<20	8(2.5)	2(0.6)	3(0.9)		3(0.9)	0.443	2(0.6)	2(0.6)	4(1.3)	0.846
20–29	94(32.2)	31(9.8)	39(12.3)	6(1.9)	45(14.2)		24(7.6)	25(7.9)	49(15.4)	
30–39	117(36.9)	27(8.5)	53(16.8)	2(0.6)	55(17.4)		37(11.7)	25(7.9)	62(19.6)	
≥40	98(30.9)	25(7.9)	48(15.2)	5(1.6)	53(16.8)		25(7.9)	20(6.2)	45(14.2)	

**Religion**										
Christian	255(80.4)	70(22.1)	108(34.1)	10(3.1)	118(37)	0.607	77(24.3)	60(18.9)	137(43.2)	0.237
Muslim	62(19.6)	16(5.0)	35(11.1)	3(0.9)	38(12.0)		11(3.5)	13(4.1)	24(7.6)	

**Education level**										
primary	96(30.3)	20(6.3)	40(12.6)	3(0.9)	43(13.5)	0.115	36(11.4)	17(5.4)	53(16.8)	0.004*
secondary	135(42.6)	39(12.3)	66(20.8)	4(1.3)	70(22.1)		30(9.5)	35(11.0)	65(19.5)	
College	67(21.1)	21(6.6)	33(10.4)	4(1.3)	37(11.7)		13(4.1)	17(5.4)	30(9.5)	
university	19(6.0)	6(1.9)	4(1.3)	2(0.6)	6(1.9)		9(2.8)	4(1.3)	13(4.2)	

**Contraceptive use**										
other	223(70.3)	66(20.8)	95(30.0)	8(2.5)	103(32.5)	0.469	62(19.6)	58(18.3)	120(37.9)	0.131
hormonal	94(29.7)	20(6.3)	48(15.1)	5(1.6)	53(16.8)		26(8.2)	15(4.7)	41(12.7)	

**parity**										
>3	68(21.5)	16(5.0)	30(9.5)	8(2.5)	38(12.0)	0.003*	22(6.9)	39(12.3)	41(39.2)	0.001**
≤3	249(78.5)	70(22.1)	113(35.6)	5(1.6)	118(37.2)		66(20.8)	34(10.7)	100(21.5)	

**Marital status**										
married	226(71.3)	55(17.4)	109(34.4)	8(2.5)	117(36.9)	0.249	62(19.6)	47(14.8)	109(34.4)	0.299
separated	32(10.1)	8(2.5)	13(4.1)	1(0.3)	14(4.4)		11(3.5)	7(2.2)	18(5.7)	
single	41(12.9)	12(3.8)	17(5.4)	2(0.6)	19(6.0)		12(3.8)	10(3.2)	22(7.0)	
divorced	6(1.9)	3(0.9)	2(0.6)	1(0.3)	3(0.9)		1(0.3)	2(0.6)	3(0.9)	
widowed	12(3.8)	8(2.5)	2(0.6)	1(0.3)	3(0.9)		2(0.6)	7(2.2)	9(2.8)	

**Number of sex** **partners**										
1	186(58.7)	43(13.6)	91(28.7)	7(2.2)	98(30.9)	0.339	52(16.4)	36(11.4)	88(27.8)	0.014*
>1	131(41.3)	43(13.6)	52(16.4)	6(1.9)	58(18.3)		36(11.4)	37(11.7)	73(23.1)	

**Total**	**317(100.0)**	**86(27.1)**	**143(45.1)**	**13(4.1)**	**156(49.2)**		**88(27.8)**	**73(23.2)**	**161(50.8)**	**0.001** *

N: negative; P: positive;

** the probability at the 0.001 level

* the probability at the 0.005 level

**d. Distribution of HPV genotypes among HIV and non HIV infected women.**

HPV type's distribution among HIV-infected women was significantly associated with residence, age, parity, family planning method and number of sex partners (Table 3).

**Table 3:** Distribution of HPV genotypes among HIV and non-HIV infected women

Category	HIV test	HPV infection													total	p-value

		High-risk HPV types					Low-risk HPV types									

		16	45	53	58	66	9	11	44	52	61	81	87	88		
Residence																
Embu	**N**	1(0.9)				1(0.9)						4(3.8)			**6(5.7)**	0.042*
	**P**	3(2.8)	1(0.9)	2(1.9)	1(0.9)	2(1.9)	1(0.9)	4(3.8)		1(0.9)		13(12.8)	1(0.9)		29(27.6)	
Isiolo	**N**	2(1.9)				3(2.8)						1(0.9)			6(5.7)	0.001**
	**P**	1(0.9)				3(2.8)		3(2.8)	1(0.9)			11(10.5)		1(0.9)	20(19.0)	
Kirinyaga	**N**											1(0.9)			1(0.9)	0.076
	**P**	2(1.9)										8(7.6)			10(9.5)	
Meru	**N**	1(0.9)		1(0.9)				1(0.9)			1(0.9)	3(2.8)			7(6.6)	0.001**
	**P**	2(1.9)										15(14.3)			17(16.2)	
T.Nithi	**N**											1(0.9)			1(0.9)	0.048*
	**P**							2(1.9)				6(5.7)			8(7.6)	

**age**																
<20	**N**															
	**P**	1(0.9)				1(0.9)						2(1.8)			4(3.8)	0.237
20–39	**N**	1(0.9)		1(0.9)		1(0.9)		1(0.9)			1(0.9)	4(3.8)			9(8.6)	
	**P**	2(1.8)	1(0.9)	1(0.9)		2(1.8)	1(0.9)	3(2.8)		1(0.9)		20(19.0)	1(0.9)		32(2.9)	0.004*
30–39	**N**	1(0.9)				1(0.9)						1(0.9)			3(2.8)	
	**P**	1(0.9)		1(0.9)		2(1.8)		5(5.8)				17(16.2)			26(24.7)	0.001**
>40	**N**	1(0.9)				1(0.9)						4(3.8)			6(5.7)	
	**P**	5(5.8)			1(0.9)	2(1.8)		1(0.9)	1(0.9)			14(13.3)		1(0.9)	23(21.7)	0.017**

**Family planning**																
hormonal	**N**	1(0.9)				1(0.9)						4(3.8)			6(5.7)	0.047*
	**P**	1(0.9)		2(1.9)		3(2.8)	1(0.9)	2(1.9)				10(9.5)			19(18.1)	
other	**N**	3(2.8)		1(0.9)		2(1.9)		1(0.9)			1(0.9)	5(4.8)			13(12.8)	0.001**
	**P**	7(6.6)	1(0.9)		1(0.9)	4(3.8)		7(6.6)	1(0.9)	1(0.9)		43(40.9)	1(0.9)	1(0.9)	67(63.8)	

**parity**																
≤3	**N**	1(0.9)				1(0.9)						2(1.9)			4(3.8)	0.001**
	**P**	2(1.9)				1(0.9)		3(2.8)	1(0.9)			8(7.6)			15(14.3)	
>3	**N**	3(2.8)		1(0.9)		2(1.9)		1(0.9)				7(6.6)			14(13.3)	
	**P**	6(5.7)	1(0.9)	2(1.9)	1(0.9)	6(5.7)	1(0.9)	6(5.7)		1(0.9)	1(0.9)	45(42.8)	1(0.9)	1(0.9)	72(68.6)	

**Number of sex partners**																
one	**N**	3(2.8)				3(2.8)						4(3.8)			10(9.5)	0.001**
	**P**	3(2.8)	1(0.9)		1(0.9)	1(0.9)		5(4.8)		1(0.9)		28(26.8)	1(0.9)	1(0.9)	42(40.0)	
>one	**N**	1(0.9)						1(0.9)			1(0.9)	5(4.8)			8(7.6)	0.001**
	**P**	5(4.8)		1(0.9)		6(5.7)	1(0.9)	4(3.8)	1(0.9)			25(23.8)			43(40.9)	

**Total**	**N**	**4(3.8)**				**3(2.8)**		**1(0.9)**			**1(0.9)**	**9(8.6)**			**18(17.1)**	**0.001** **
	**P**	**8(7.6)**	**1(0.9)**	**1(0.9)**	**1(0.9)**	**7(6.6)**	**1(0.9)**	**9(8.6)**	**1(0.9)**	**1(0.9)**		**53(50.5)**	**1(0.9)**	**1(0.9)**	**85(80.9)**	

N: negative; P: positive

** the probability at the 0.001 level

* the probability at the 0.005 level

**e. Association of cervical cytology with other clinical reproductive health ailments**

**Figure 2:**
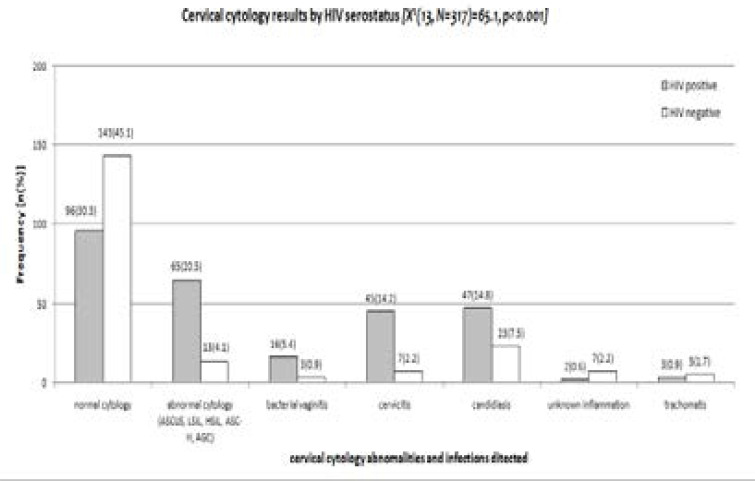
Cervical cytology results

A total of 96(30.3%) HIV-infected women had normal cytology as compared to 143(45.1%) HIV-uninfected, whereas 65(20.5%) HIV-infected women had abnormal cytology results compared with 13(4.1%) HIV-uninfected (p=0.001) (Figure 2).

**f. Association of HPV genotypes with cervical histological results among women HIV infected and non-infected women**

HPV genotypes detected by cervical histological results were: CIN117(5.4%), CIN216(5.0%, CIN36(1.8%) and invasive cancer5(1.5%) (p<0.001) (Table 4).

**Table 4:** HPV types detected by Pap smear results

HIV status	HPV type of infection	Normal	§Histological analysis of abnormal cytology samples (n=78)						

			ASCUS	CIN 1	CIN2	CIN3	ICC	Total	p-value
**Negative**	**Single type**								
	HPV 81	3(0.9)	1(0.3)	2(0.6)	2(0.6)	1(0.3)		9(2.7)	0.001*
	**Multiple types**								
	HPV 16,66	2(0.6)					1(0.3)	3(0.9)	
	HPV 11,16,53,81,61	1(0.3)						1(0.3)	
	HPV Positive	6(1.9)	1(0.3)	2(0.6)	2(0.6)	1(0.3)	1(0.3)	13(4.1)	
	HPV Negative	137(43.2)	6(1.8)					143(45.1)	
	**Total (HPV** **positive** **and negative)**	**143(45.1)**	**7(2.1)**	**2(0.6)**	**2(0.6)**	**1(0.3)**	**1(0.3)**	**156(49.2)**	
**Positive**	**Single type**								
	HPV 11	2(0.6)	1(0.3)	2(0.6)	1(0.3)	1(0.3)		7(2.2)	0.001*
	HPV 16	1(0.3)			1(0.3)		1(0.3)	3(0.9)	
	HPV 66				1(0.3)			1(0.3)	
	HPV 81	21(6.6)	5(1.6)	12(3.8)	6(1.9)	4(1.2)	3(0.9)	51(16.1)	
	**Multiple types**								
	HPV11, 66				1(0.3)			1(0.3)	
	HPV 81,44				1(0.3)			1(0.3)	
	HPV 81, 88				1(0.3)			1(0.3)	
	HPV 9,53				1(0.3)			1(0.3)	
	HPV 16,58				1(0.3)			1(0.3)	
	HPV 16,66	3(0.9)	1(0.3)					4(1.2)	
	HPV 66,53			1(0.3)				1(0.3)	
	HPV 11,45,52,87	1(0.3)						1(0.3)	
	HPV Positive	28(8.8)	7(2.1)	15(4.7)	14(4.4)	5(1.6)	4(1.2)	73	
	HPV Negative	68(21.4)	20(6.3)					88(27.8)	
	**Total (HIV positive** **and negative)**	**96(30.3)**	**27(8.5)**	**15(4.7)**	**14(4.4)**	**5(1.6)**	**4(1.2)**	**161(50.8)**	
**Total (HIV positive and** **negative)**		**239(75.4)**	**34(10.7)**	**17(5.4)**	**16(5.0)**	**6(1.8)**	**5(1.5)**	317(100.0)	

ASCUS: Atypical Cells of Unknown Significance; CIN: Cervical Intraepithelial Neoplasia; ICC: Invasive cervical cancer; *: the probability at the 0.001 level, § abnormal cytology samples: Atypical Cells of Unknown Significance (ASCUS), Low-grade squamous intraepithelial lesion (LSIL), High-grade squamous intraepithelial lesion (HSIL), atypical squamous cells, cannot exclude HSIL (ASC-H) or Atypical glandular cells (AGC)].

**g. Distribution of HPV genotypes among HIV and non-HIV infected women with cervical dysplasia**

**Figure 3:**
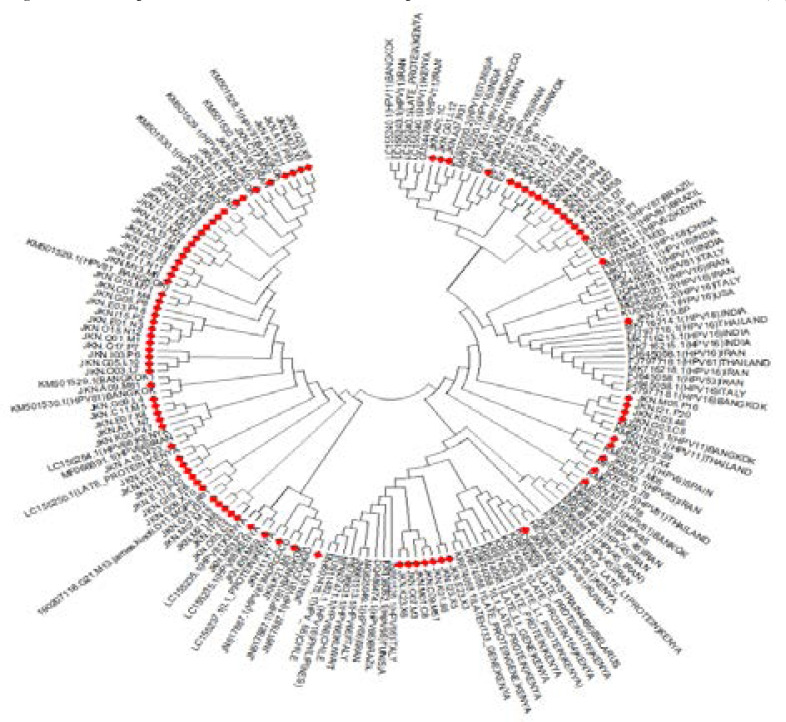
Phylogenetic analysis of HPV detected in eastern Kenya.

Phylogenetic tree of HPV samples marked in red aligned against the representation of the different HPV genotypes distributed worldwide. Most HPV81 clustered with those cases detected in Bangkok, Morocco and Thailand while HPV66 clustered with cases reported in Tunisia, Morocco, Iran and India (Figure 3).

## Discussion

This study established an overall HPV infection-rate of 27.12% among HIV-infected (23.03%) and HIV-non infected (4.1%) women. It disagrees with the overall HPV infection rate of 40.0%1 in Kenya^11^ 13.7% in Ethiopia^12^. Embu and Meru Counties had the highest overall HPV infection rate among HIV-infected women. This is indicator of the expected high burden of cervical neoplasia in the region. Women aged below 35 years had a high rate of mixed HPV genotypes and a significant association between HIV infection and abnormal cytology outcome which agrees with published observations^1^, ^5^,^7^. A possible explanation is that HIV infection may facilitate the progression of HPV infection to cancer in young women, and an inverse relationship of highrisk HPV prevalence and age has been described^4^,^11^,^13^.

Single HPV type's infection in CIN1+ showed diversity compared with multiple HPV type's infection by HIV-infection. HPV44(α10), HPV58, HPV81, and HPV88 do not feature in many studies as potential oncogenic types, and their dominance in cervical dysplasia increases with HIV infection^1^,^8^,^18^,^19^ as seen in this study. Members of (α7) and (α9) dominate malignant tissues coding for a hydrophobic E5, hence considered oncogenic. Furthermore, following HPV infection, antibodies cross-reaction is achieved intraspecies unlike across species, and the risk of contracting the same HPV strain is reduced significantly. Antibodies against HPV16 cross-react HPV52 and HPV58 (α9) species, antibodies against HPV18 cross-react with HPV66, while HPV53 (α7) and HPV81 cross-react with HPV6120.

This study and literature data in Kenya establish the predominance of mixed HPV types among HIV-infected women other than those included in the current bivalent (against HPV16 and 18) and quadrivalent (against HPV6, 11, 16 and 18) vaccine^4^,^18^,^20^ in Kenya.

Literature data have shown limited efficacies of the HPV vaccines against non-vaccine HPV types^18^,^19^,^20^. Hence, there is need for increased effective HPV vaccination coverage among HIV-infected women with a diversified nonavalent (9vHPV) vaccine^21^,^22^,^23^,^24^ that protects against most types detected in this study.

The phylogenetic trees illustrated that single or multiple HPV types infected each participant as shown by 15 participants. Another reason is that HPV genes are replicated by the host replication machinery suggesting that a very low human autosomal-like mutation rate would be operating as established in another.

## Limitation

The study group is a minimal presentation and data presented, cannot be generalized as exact outcome if all women were sampled.

## Conclusion

There were more mixed lr/hrHPV genotypes associated with cervical dysplasia among HIV-infected than non-HIV uninfected women attending reproductive health facilities in Eastern Kenya. The finding adds to the pool of knowledge the epidemiological data required in determining the population at risk of cervical cancer.

## Data Availability

The datasets are available from the corresponding author on reasonable request.
